# FTIR Investigation of Secondary Structure of Reteplase Inclusion Bodies Produced in *Escherichia coli* in Terms of Urea Concentration

**DOI:** 10.22037/ijpr.2020.1101092

**Published:** 2020

**Authors:** Sajjad Naeimipour, Seyed Abbas Shojaosadati, Ahmad Fazeli

**Affiliations:** *Biotechnology Group, Chemical Engineering Department, Tarbiat Modares University, Tehran, Iran.*

**Keywords:** Fourier transformed infrared technique (FTIR), Non-classical inclusion body, non-denaturing solubilization, Reteplase

## Abstract

Recent studies suggest that reducing the induction temperature would improve the quality of some recombinant inclusion bodies (IB) by providing a native-like secondary structure and leading to an improvement in protein recovery. This study focused on optimizing the solubilization condition of Reteplase, a recombinant protein with 9 disulfide bonds. The influence of lowering induction temperature and urea concentration was investigated on the secondary structure of the recombinant protein through FTIR analysis. Induction temperature reduction decreased the percentage of helixes and loops from 49 to 8. In addition, FTIR spectroscopy corroborates the drastic impact of this parameter on Reteplase secondary structure. Even though lowering urea concentration tripled the solubility of IBs expressed at lower induction temperature, the final yield is still quite low to be considered as optimum. On the other hand, the percentage of *beta* strands and turns in secondary structure of dissolved proteins were proportional to urea concentration. Therefore, in case of Reteplase, protein expression at low temperature (25 °C) was not efficient to improve the protein recovery yield. Future studies need to focus on using other methods of solubilizing IBs to improve protein recovery.

## Introduction

Overexpression of heterologous proteins in *Escherichia coli* leads to the formation of inclusion bodies (IB) (1, 2). Several factors, including the selection of an appropriate host strain, using an optimized genetic code, production at low temperatures and an appropriate medium composition can affect the activity, as well as physical and structural properties of inclusion bodies (1, 2). 

Several reports indicated that the expression of recombinant proteins at low temperatures could lead to the formation of biologically active aggregates, namely non-classical inclusion bodies (1). Non-classical IBs are soluble in mild solubilizing agents whereas high agent concentration is required to solubilize the classical IBs (1, 3 and 4). Based on structural evaluations, non-classical IBs contain some characteristics of native-like secondary structure (1, 3). 

Inclusion bodies are usually solubilized in high concentration of denaturants, such as urea or guanidine hydrochloride while being treated with reducing agents (5, 6). Generally, high concentrations of denaturants might deteriorate the IBs structure. Some studies demonstrate that by using mild denaturants, such as 0.2% N-lauroyl sarcosine, organic solvents (*e.g.* n-propanol) and aggregation suppressors (*e.g.* Arginine), higher portion of the native-like secondary structure of IBs could be preserved during solubilization (3, 5 and 7-9).

During the last 50 years, the underlying mechanisms of salting-out and denaturation of proteins have been investigated by many researchers. It has been reported that proteins prefer to interact with denaturant molecules (10). According to these findings, amino acids buried within the protein in native state will be exposed to the solvent in presence of urea as a result of the affinity of protein molecules for urea molecules. Therefore, increasing urea concentration would denature more proteins since more amino acids become exposed to the urea molecules (11, 12). 

In the present study, the effect of urea concentration on structural characteristics of Reteplase IBs has been thoroughly investigated through FTIR technique for the first time up to the extent of our knowledge. The protein is a truncated form of the tissue-plasminogen activator (tPA) and used for treating acute myocardial infarction. Reteplase contains 9 disulfide bonds and its overexpression in *Escherichia coli (E. coli) *results in formation of inactive IBs (13). Previous studies have mostly been directed to understanding of the behavior of proteins towards reducing induction temperature or using non-denaturing solubilization buffers, while none has focused on commercial proteins such as Reteplase and how these production conditions could affect the final yield. By focusing on induction temperature and denaturant concentration, we tried to optimize recombinant Reteplase production using *Escherichia coli* in terms of conditions best preserving the secondary structure and increasing the refolding yield.

## Experimental

*Chemicals and Reagents*

Two different types of Reteplase IBs were generously donated by Zist Darou Danesh Co. (Tehran, Iran). Both Reteplase IBs were expressed in *Escherichia coli* BL21, one at 37 °C and the other at 25 °C. These IBs were purified and washed several times according to the Zist Darou Danesh instructions and the final pellet was used for this study. Ethylenediaminetetraacetic acid

(EDTA, Sigmaldrich, 60-00-4), Tris(hydroxymethyl)aminomethane (Tris-base, Sigma-Aldrich, 77-86-1), 1,4-Dithiothreitol (DTT, Sigma-Aldrich, 3483-12-3), Urea (Sigma-Aldrich, 57-13-6), Coomassie Brilliant Blue G-250 (Sigma-Aldrich, 6104-58-1), Methanol (Sigma-Aldrich, 67-56-1), Phosphoric acid (Sigma-Aldrich, 7664-38-2), Bovine serum albumin (BSA, Sigma-Aldrich, 9048-46-8) were used in this research.

Freeze dryer (Christ, alpha1-2LDplus, Germany), and FTIR (PerkinElmer Frontier, USA) were the main equipment recruited in this study.

*Solubilization of Reteplase IBs*

Experiments were performed in triplicate for each sample and the average values were considered for data analysis. A 300 mg sample (wet weight) of IBs was soaked in a 2 mL solubilization buffer, which prepared accordingly. The solubilization buffer is composed of 50 mM Tris–base, 1 mM EDTA, 100 mM DTT, and different concentrations of urea, including 2, 4, 6, 8 and 10 M (7). According to the isoelectric point of the protein, the pH of the solubilization buffer was adjusted to 8. IBs were stirred gently by magnetic stirrer overnight at ambient temperature. Finally, samples were centrifuged at 12000 rpm and 4 °C for 10 min. The supernatant was separated and used for further evaluations. 

*Total Protein Analysis*

Total protein concentration was determined according to the Bradford method (14). Bovine serum albumin was used as a standard. The percentage of solubility was calculated as follows:

$\mathrm{Solubility\%}=\frac{\mathrm{Total Protein Concentration}\times \mathrm{Sample Volume}(\mathrm{in mL})}{\mathrm{Dry Weight of IB}}$

Equation 1

*Fourier Transformed Infrared Spectroscopy (FTIR)*

Solubilized proteins were precipitated by adding saturated ammonium sulfate to the samples and centrifuging at 12000 rpm and 4 °C for 10 min to separate the precipitated proteins (3). The pellets were washed three times with distilled water, and the washed precipitated proteins were then dried by freeze dryer for 10 h prior to analysis by FTIR.

Each participated sample was mixed with ground KBr and pressed to form a pellet. The FTIR data were collected with 64 scans and a resolution of 4 cm^-1^, while dry nitrogen was purged into the instrument. The results in the mid-infrared region (from 4000 to 400 cm^-1^), amide I (1600 to 1700 cm^-1^) and amide II (1480 to 1575 cm^-1^) were compared and subjected to some mathematical resolution enhancement processes. 

*FTIR data mathematical enhancement*

The second derivative of results in amide I region, as a common resolution enhancement method, were used to analyze the secondary structure of proteins (15). Based on the data obtained from second derivatives of spectra, curve fitting was performed using a Matlab code (18). For each peak a Gaussian curve was fitted. The accepted error (sum of squares of the deviations normalized by the variance of the count) for fitting the curve to the results was considered to be less than 4%. The interpretation of these results has been based on preceding studies on the secondary structure of proteins by the same method (1, 3, 4 and 7). 

## Results

*IBs solubility*

**Figure 1:**
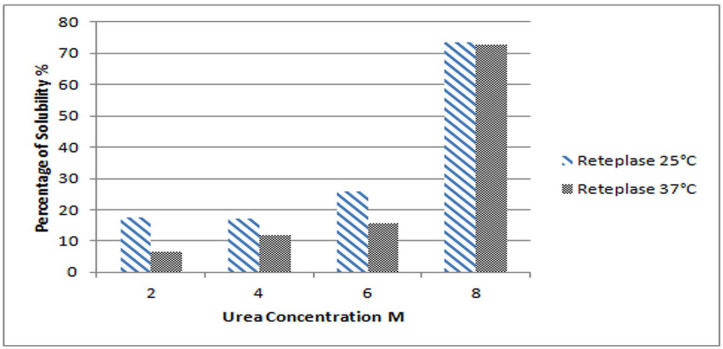
Solubility of Reteplase IBs expressed at 25 °C and 37 °C in solutions containing 2-8 M urea

To optimize the best condition for IBs solubilization, different concentrations of urea were examined in terms of IBs solubility whose production was induced at two different temperatures. Figure 1 illustrates the solubility of Reteplase IBs produced at two different induction temperatures of 25 and 37 °C as a function of urea concentration. The reduction of Reteplase expression temperature has a great impact on the solubility of IBs in lower urea concentration. However, the final percentage of protein solubilized in 8 M urea is almost equal for both expression conditions. This is in agreement with previous studies and suggests that reducing the induction temperature leads to the formation of IBs with higher solubility in mild detergent concentrations (2-4). 

*Impact of induction temperature on IBs secondary structure*

**Figure 2:**
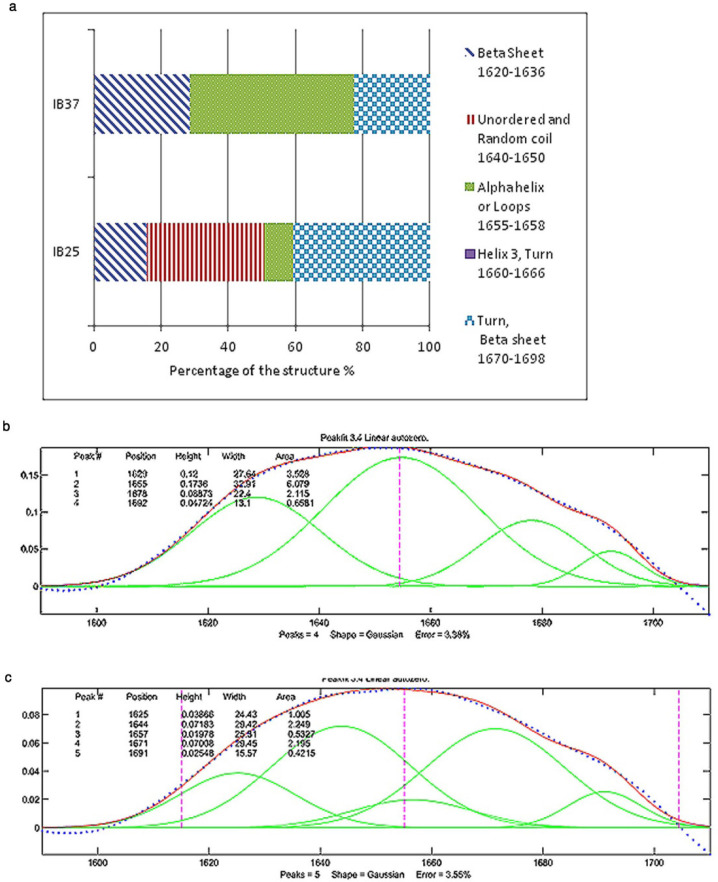
(a) Percentages of different secondary structures in the inclusion bodies of Reteplase expressed at 25 °C and 37 °C based on the curve fitting of FTIR results. IB 37 and IB 25 represent Reteplase IBs expressed at 37 °C and 25 °C. (b) FTIR results for Reteplase IB expressed at 37 °C. (c) FTIR results for Reteplase IB expressed at 25 °C

In recombinant protein production, it is believed that induction temperature is one the main factors with influence on the formation of native-like secondary structures in IBs (3). The reduction of induction temperature to 25 °C had a drastic effect on the secondary structure of Reteplase inclusion bodies (Figure 2). The percentage of regions pertaining to the immobilized structures, such as helices and loops (1655-1658 cm^-1^), plunged to about 8% by decreasing the temperature (16). As shown in Figure 2, the percentage of β-sheets that occurred in the lower frequencies of the amide-I region (1620-1636 cm^-1)^ is higher for IBs induced at 37 °C compared to 25 °C, which indicates that these IBs are more compact than those produced at lower temperature (16). Additionally, a recent study modeled the secondary structure of HV12p-rPA, which is a novel hybrid (HV12p-rPA) comprised of the C-terminal 12 residues of hirudin-PA (HV12p) and Reteplase (rPA). The structure of this hybrid protein is beta strands and unordered structures rich (19). Based on this model the secondary structure of IBs produced at 25 °C seems to be close to the native state of Reteplase. 

*Impact of urea on the secondary structure of solubilized proteins*

**Figure 3:**
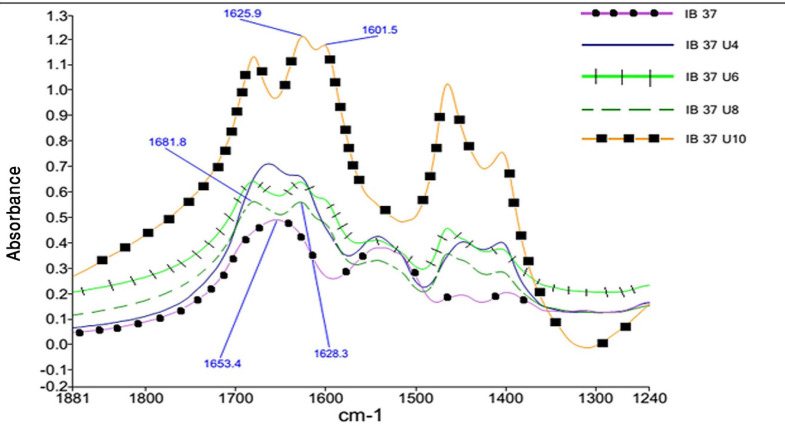
Changes in amide I region absorbance of Reteplase expressed at 37 °C as the concentration of Urea changes. Black circle Dry IB, Black square IB dissolved in urea 10 M, Dashed line IB dissolved in urea 8 M, Crossed line IB dissolved in urea 6 M, Simple line IB dissolved in urea 4 M

As the concentration of urea in the solution increases, the single peak in amide I band region splits into three distinct peaks. Figure 3 shows this phenomenon for Reteplase IBs expressed at 37 °C. This figure *per se* is representative of the drastic changes in the structure of IBs as dissolved in urea. This was also true for IBs produce at 25 °C.

**Figure 4:**
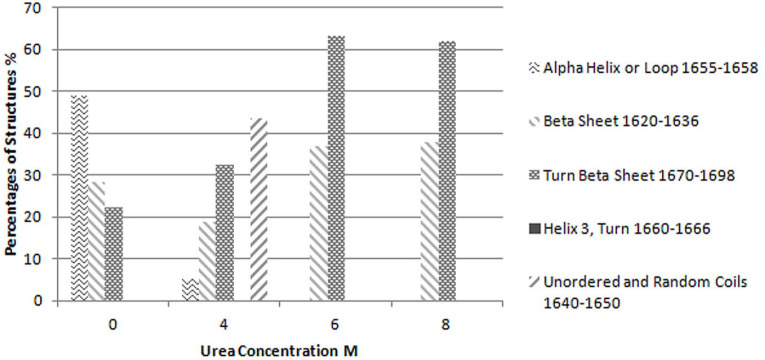
Percentages of each structure evaluated based on the curve fitting results for Reteplase IBs expressed at 37 °C and dissolved in different concentrations of urea

**Figure 5:**
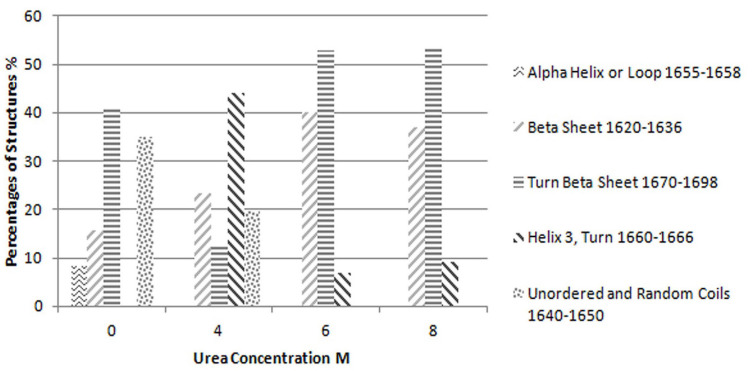
Percentages of each structure evaluated based on the curve fitting results for Reteplase IBs expressed at 25 °C and dissolved in different concentrations of urea

Considering this figure and without need of any resolution enhancement technique, it is clear that protein structure at 10 molar Urea concentration was completely unfolded and the main structures are beta strands. However, by applying resolution enhancement techniques, the trend of these changes was meticulously investigated. Consequently, as shown in Figure 4, immobilized-secondary structures, such as helices and loops are very sensitive toward urea concentration, and are more vulnerable than other structures. According to these results, it appears that the percentage of beta strands and turns increases with urea concentration (Figures 4 and 5) and immobilized-secondary structure vanishes at higher urea concentrations. In the Figure 3, at 10 molar urea concentration peaks shifted to the both sides of the amide I region which is pertaining to beta sheets and turns. 

## Discussion

The high-level expression of heterologous proteins in *E. coli* leads to the formation of IBs. As reported in previous studies, reduction of protein expression temperature forms non-classical IBs with the native-like secondary structure. While comparing the solubility of IBs in urea as a function of temperature, it was observed that lower temperature of induction tripled the solubility of the resultant IBs in 2 M urea. The finding of increased solubilization of IBs expressed at low induction temperature in mild denaturant concentration is in accordance with previous studies (2-4). However, even at lower induction temperature the solubility of Reteplase in 2 M urea is quite low, less than 20%. 

Urea is an inexpensive versatile reagent and is commonly used as a denaturant for commercial products. The effect of different concentrations of urea on the secondary structure of Reteplase was investigated using FTIR technique. According to the FTIR results, the structure of IBs produced at lower temperature is less compact, and this could be attributed to the higher solubility of these IBs in lower urea concentration.

According to the Tanford explanation, when a protein unfolds, the buried peptides become exposed to the solvent molecules (17). Timsheff *et al.* showed that urea molecules have a greater tendency toward protein molecules in comparison with water molecules. Therefore, as the concentration of urea increases, the structure in which mode peptides are exposed to solvent, is induced (11). The results of the current study are in line with the points discussed. By increasing the urea concentration in the environment, the percentage of beta strands and turns increases and immobile structures such as helices and loops vanish. Immobile structures limit the flexibility of the protein structure and make it difficult for the solvent molecules to reach the buried peptides, thus, as urea concentration is increased, the structure of the protein changes into more flexible and exposed structures like beta strands and turns. This change in the structure of the protein lets the buried peptides become exposed to the solvent.

Hence, it could be expected for those IBs that immobile structures form most of their body (which would be as a consequence of the protein nature) to experience a dramatic change in their structure at higher concentrations of urea, and also they might not be solubilized in lower concentrations.

## Conclusion

The results of the present work are in congruent with previous studies on the effect of induction temperature on IBs quality. Reteplase IBs solubility is directly proportional to urea concentration. Consequently, IBs production at low temperature does not necessarily leads to obtain higher yield in protein refolding, even though the IB has non-classic like characteristic. Nevertheless, owing to the key role of beta structures and turns in reteplase solubilization, it could be a good research topic for the further investigations that the production of proteins with higher portion of beta structures in their native state can be improved by reduction of the induction temperature and solubilization of IBs in lower concentrations of urea.
